# Analysis of the Expression and Prognostic Value of MSH2 in Pan-Cancer Based on Bioinformatics

**DOI:** 10.1155/2021/9485273

**Published:** 2021-11-23

**Authors:** Wenli Qiu, Ke Ding, Lusheng Liao, Yongchang Ling, Xiaoqiong Luo, Junli Wang

**Affiliations:** ^1^Medical Reproduction Center, Affiliated Hospital of Youjiang Medical University for Nationalities, Baise, Guangxi, China; ^2^Department of Sports Medicine, Affiliated Hospital of Youjiang Medical University for Nationalities, Baise, Guangxi, China; ^3^School of Medical Laboratory Science, Youjiang Medical University for Nationalities, Baise, Guangxi, China; ^4^Department of Obstetrics, Affiliated Hospital of Youjiang Medical University for Nationalities, Baise, Guangxi, China

## Abstract

**Background:**

MutS homolog 2 (MSH2), with the function of identifying mismatches and participating in DNA repair, is the “housekeeping gene” in the mismatch repair (MMR) system. MSH2 deficiency has been reported to enhance cancer susceptibility for the association of hereditary nonpolyposis colorectal cancer. However, the expression and prognostic significance of MSH2 have not been studied from the perspective of pan-cancer.

**Methods:**

The GTEx database was used to analyze the expression of MSH2 in normal tissues. The TCGA database was used to analyze the differential expression of MSH2 in pan-cancers. The prognostic value of MSH2 in pan-cancer was assessed using Cox regression and Kaplan-Meier analysis. Spearman correlations were used to measure the relationship between the expression level of MSH2 in pan-cancer and the level of immune infiltration, tumor mutational burden (TMB), and microsatellite instability (MSI).

**Results:**

MSH2 is highly expressed in most type of cancers and significantly correlated with prognosis. In COAD, KIRC, LIHC, and SKCM, the expression of MSH2 was significantly positively correlated with the abundance of B cells, CD4+ T cells, CD8+ T cells, dendritic cells, macrophages, and neutrophils. In THCA, MSH2 expression correlated with CD8+T Cell showed a significant negative correlation. MSH2 had significantly negative correlations with stromal score and immune score in a variety of cancers and significantly correlated with TMB and MSI of a variety of tumors.

**Conclusions:**

MSH2 may play an important role in the occurrence, development, and immune infiltration of cancer. MSH2 can emerge as a potential biomarker for cancer diagnosis and prognosis.

## 1. Introduction

Cancer has seriously endangered global public health. The incidence and mortality of cancer are increasing rapidly every year, which has gradually become the primary killer threatening human health [1, 2]. Despite continuing improvements in diagnosis and treatment methods of cancers, a series of problems such as cancer recurrence and drug resistance still lead to the low survival [3]. Therefore, it is urgent to find novel methods for cancer diagnostics and treatments. With the continuous development and improvement of public databases for example The Cancer Genome Atlas (TCGA), new immunotherapy targets have been discovered through pan-cancer expression analysis of genes and evaluation of their relationship with clinical prognosis and immunity [4]. Autophagy-related protein 5 is a protein related to autophagosome formation. Studies using public database analysis have found that autophagy-related protein 5 plays an important role in tumor metabolism and tumor immunity through pan-cancer analysis and is a promising tumor predictive biomarker in most solid tumors [5].

MutS homolog 2 (MSH2) is homologous to the *E. coli* MutS gene and participates in DNA mismatch repair (MMR) [6, 7]. Human MSH2/6 can form a complex with BLM-p53-RAD51 in response to DNA damage repair [8]. When DNA is damaged, MSH2 promotes cell apoptosis by regulating ATR/Chk2/p53 signal transduction [9]. In addition, MSH2 is not only closely associated with the autophagy pathway. MSH2 deficiency can also cause accelerated telomere shortening in normal human cells [10, 11].

MSH2 gene is intimately linked to the occurrence and development of cancer, whose expression ratios is different in various types of malignant lymphoproliferative diseases derived from B cells [12]. MSH2 missense mutations affect splicing, which may regulate the occurrence and development of cancer in a tissue-specific manner [13]. MSH2-deficient tumor cell lines have lost most of the ability to accurately repair plasmid DNA double-strand breaks through homologous recombination and increased susceptibility to cancer by promoting deletions or insertion mutations associated with DNA double-strand break repair [14]. Recent studies have indicated that the MSH2 is closely related to the occurrence of Lynch syndrome (Lynch), whose new splicing site mutation (c.1661+2 T>G) can cause Lynch [15, 16]. Lynch is an autosomal dominant genetic disease. Lynch patients have a significantly increased risk of breast and multiple gastric cancer [17, 18]. It is reported that the abnormal expression of MSH2 is related to cancers such as oral squamous cell carcinoma, primary prostate cancer, breast cancer, and gastric cancer [18–21]. However, MSH2 is currently being investigated in specific cancer types rather than analyzed from the perspective of pan-cancer. In this study, we evaluated the expression and prognostic value of MSH2 in pan-cancer and analyzed the association in the MSH2 expression levels and tumor microenvironment, tumor mutational burden (TMB), and microsatellite instability (MSI) in 33 cancer types.

## 2. Materials and Methods

### 2.1. Sample Information

The Cancer Genome Atlas (TCGA; https://portal.gdc.cancer.gov/) contains clinical data of 33 cancer types, allowing cancer researchers to search and download cancer data for analysis. Download data of 33 different cancer types in the TCGA database through UCSC Xena (https://xena.ucsc.edu/). Genotype-Tissue Expression (GTEx; https://gtexportal.org/) studied more than 7,000 autopsy samples from 449 healthy human donors, covering 44 tissues. We obtained 31 different normal tissue MSH2 expression matrix and clinical information data through GTEx database. The TIMER database (https://cistrome.shinyapps.io/timer/) is a comprehensive resource for systematically analyzing the immune infiltration of different cancer types. It can analyze the correlation between the expression of the MSH2 gene and the abundance of immune infiltration in pan-cancer. We downloaded score data of six immune infiltrating cells from 33 cancers from the timer database and analyzed the correlation between MSH2 gene expression and the score of these immune cells. The full name and abbreviation of cancer are given as follows: adrenocortical carcinoma (ACC); bladder urothelial carcinoma (BLCA); breast invasive carcinoma (BRCA); cervical squamous cell carcinoma and endocervical adenocarcinoma (CESC); cholangiocarcinoma (CHOL); colon adenocarcinoma (COAD); lymphoid neoplasm diffuse large B-cell lymphoma (DLBC); esophageal carcinoma (ESCA); glioblastoma multiforme (GBM); head and neck squamous cell carcinoma (HNSC); kidney chromophobe (KICH); kidney renal clear cell carcinoma (KIRC); kidney renal papillary cell carcinoma (KIRP); acute myeloid leukemia (LAML); brain lower grade glioma (LGG); liver hepatocellular carcinoma (LIHC); lung adenocarcinoma (LUAD); lung squamous cell carcinoma (LUSC); mesothelioma (MESO); ovarian serous cystadenocarcinoma (OV); pancreatic adenocarcinoma (PAAD); pheochromocytoma and paraganglioma (PCPG); prostate adenocarcinoma (PRAD); rectum adenocarcinoma (READ); sarcoma (SARC); skin cutaneous melanoma (SKCM); stomach adenocarcinoma (STAD); testicular germ cell tumors (TGCT); thyroid carcinoma (THCA); thymoma (THYM); uterine corpus endometrial carcinoma (UCEC); uterine carcinosarcoma (UCS); uveal melanoma (UVM).

### 2.2. Expression Analysis of MSH2 Gene in Pan-Cancer

The differential expression of MSH2 in tumor and adjacent normal tissues was analyzed by Wilcoxon's test. The R package “ggpubr” was used to visualize pictures (^∗∗∗^*P* < 0.001; ^∗∗^*P* < 0.01; ^∗^*P* < 0.05).

### 2.3. Survival Analysis of MSH2 Gene in Pan-Cancer

Each sample downloaded from the TCGA database extracts survival-related data, was selected overall survival (OS) to study the relationship between MSH2 expression and patient survival, and analyzed by univariate survival to study the relationship between MSH2 expression and patient survival. According to the median value of MSH2 expression level, patients were divided into high-expression group and low-expression group. The Kaplan-Meier method was used to compare the survival rate of patients in the groups mentioned above. The R packages “survival” and “forestplot” were used to draw forest plots. Kaplan-Meier curves were drawn by the R package “survival” and “survminer.”

### 2.4. The Relationship between MSH2 Gene Expression and Immune Cells

The TIMER database “Gene” module was used to evaluate the correlation between the expression of MSH2 and the level of immune infiltrating cells in pan-cancer. Six types of immune infiltrating cells include B cells, CD4+ T cells, CD8+ T cells, dendritic cells, macrophages, and neutrophils. Use R-package “estimate” to calculate the immune score and stromal score in each tumor sample calculated by R-package “estimate.”

### 2.5. Association Analysis of MSH2 Gene Expression with Tumor Mutation Burden and Microsatellite Instability

Tumor mutation burden (TMB) refers to the number of base mutations per million bases. The Spearman method was used to calculate the correlation between TMB and MSH2 expression. Microsatellite instability (MSI) refers to the phenomenon that new microsatellite alleles appear at a certain microsatellite site in tumors due to the insertion or deletion of repeat units compared with normal tissues. The Spearman method was used to calculate the correlation between MSI and MSH2 expression. R package “fmsb” was applied for image visualization (^∗∗∗^*P* < 0.001; ^∗∗^*P* < 0.01; ^∗^*P* < 0.05).

### 2.6. Statistical Analysis

All the data of gene expression were normalized by log2 transformation. The differential expression of MSH2 in pan-cancer was tested by Wilcox test. The Kaplan-Meier curve and Cox proportional hazards model were used for survival analysis. The Spearman method was used to study the correlation between two variables. *P* value < 0.05 was considered as significant. The visualization of the data is processed by R software (version 4.1.0).

## 3. Results

### 3.1. MSH2 Is Highly Expressed in Pan-Cancer

**Figure 1:**
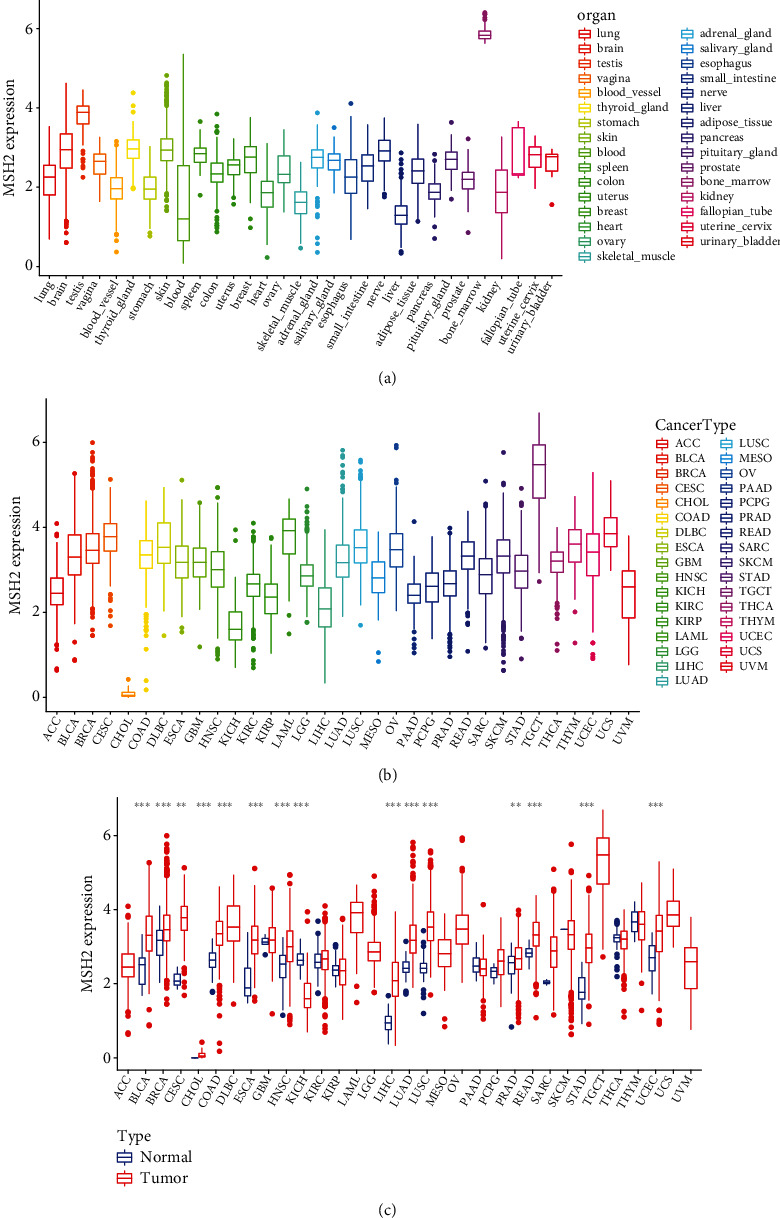
The expression of MSH2 in pan-cancer. (a) The expression of MSH2 in normal tissues. (b) The expression of MSH2 in tumor tissues. (c) Differential expression of MSH2 in normal tissues and tumor tissues. ^∗^*P* < 0.05, ^∗∗^*P* < 0.01, ^∗∗∗^*P* < 0.001.

The MSH2 expression level in bone marrow tissue was the highest among 31 kinds of normal tissues through database search of the GTEx, while lower in most other normal tissues (Figure 1(a)). Subsequently, we evaluated the expression level of MSH2 in 33 cancer types in the TCGA database. The results showed that MSH2 was widely expressed in all cancer types. Among them, MSH2 expression was highest in TGCT and lowest in CHOL (Figure 1(b)). Compared with the corresponding normal tissues based on the TCGA database, MSH2 is significantly higher expressed in BLCA, BRCA, CESC, CHOL, COAD, ESCA, HNSC, LIHC, LUAD, LUSC, PRAD, READ, STAD, and UCEC, while significantly lower expressed only in KICH (Figure 1(c)).

### 3.2. The Prognostic Value of MSH2 in Pan-Cancer

**Figure 2:**
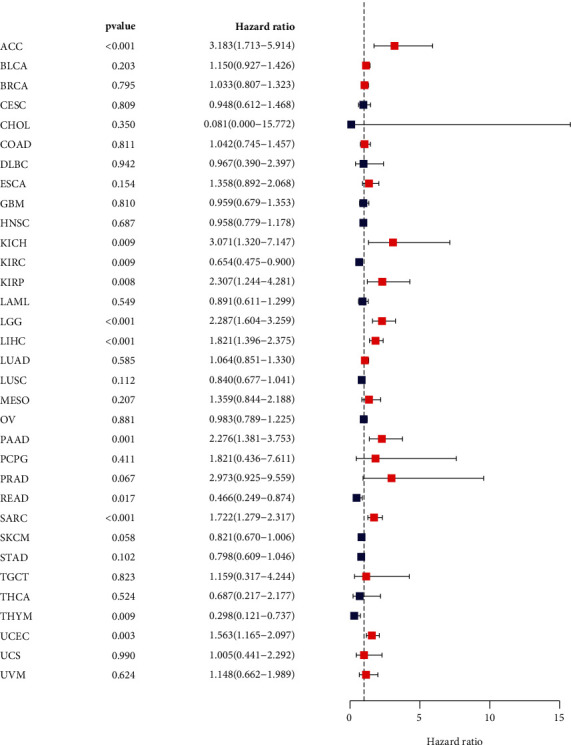
Forest plot of the correlation between MSH2 expression level and overall survival in 33 tumor types.

**Figure 3:**
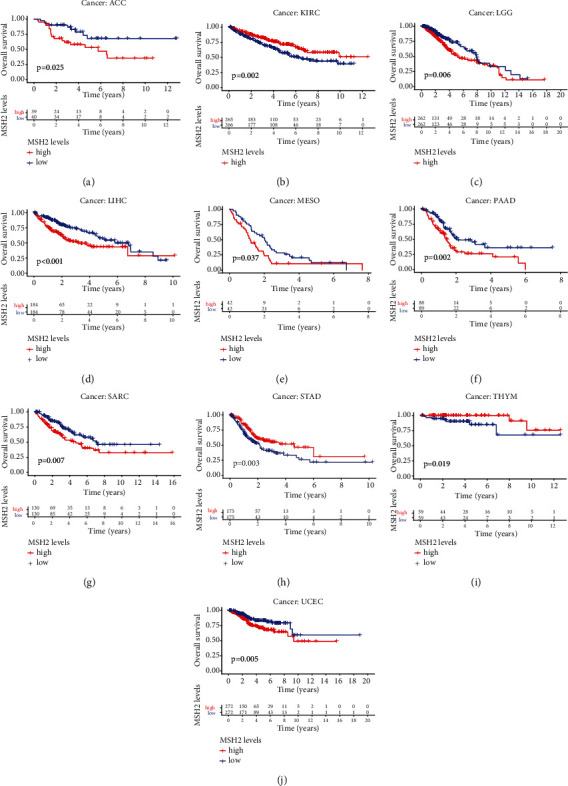
Kaplan-Meier OS curves of MSH2 expression in the ten most significantly associated tumors. (a) KM curves of high and low MSH2 expression in ACC patients. (b) KM curves of high and low MSH2 expression in KIRC patients. (c) KM curves of high and low MSH2 expression in LGG patients. (d) KM curves of high and low MSH2 expression in LIHC patients. (e) KM curves of high and low MSH2 expression in MESO patients. (f) KM curves of high and low MSH2 expression in PAAD patients. (g) KM curves of high and low MSH2 expression in SARC patients. (h) KM curves of high and low MSH2 expression in STAD patients. (i) KM curves of high and low MSH2 expression in THYM patients. (j) KM curves of high and low MSH2 expression in UCEC patients.

Univariate Cox regression analysis was used to evaluate the correlation between MSH2 expression levels in 33 different tumor types in the TCGA database and the overall survival (OS) of patients. Forest plots in 33 different types of tumors showed that the expression of MSH2 in ACC (HR = 3.183, *P* < 0.001), KICH (HR = 3.071, *P* = 0.009), KIRC (HR = 0.654, *P* = 0.009), KIRP (HR = 2.307, *P* = 0.008), LGG (HR = 2.287, *P* < 0.001), LIHC (HR = 1.821, *P* < 0.001), PAAD (HR = 2.276, *P* = 0.001), READ (HR = 0.466, *P* = 0.017), SARC (HR = 1.722, *P* < 0.001), THYM (HR = 0.298, *P* = 0.009), and UCEC (HR = 1.563, *P* = 0.003) was significantly correlated with overall survival. MSH2 was a high-risk gene in ACC, KICH, KIRP, LGG, LIHC, PAAD, SARC, and UCEC; however, MSH2 was a low-risk gene in KIRC, READ, and THYM (Figure 2). Kaplan-Meier survival analysis also demonstrated that among patients with KIRC (*P* = 0.002), STAD (*P* = 0.003), and THYM (*P* = 0.019), those with high levels of MSH2 had longer survival times, while in patients with ACC (*P* = 0.025), LGG (*P* = 0.006), LIHC (*P* < 0.001), MESO (*P* = 0.037), PAAD (*P* < 0.002), SARC (*P* = 0.007), and UCEC (*P* = 0.005), high MSH2 expression was associated with poor OS (Figure 3).

### 3.3. MSH2 Is Associated with Tumor Immune Infiltrating Cells in Pan-Cancer

**Figure 4:**
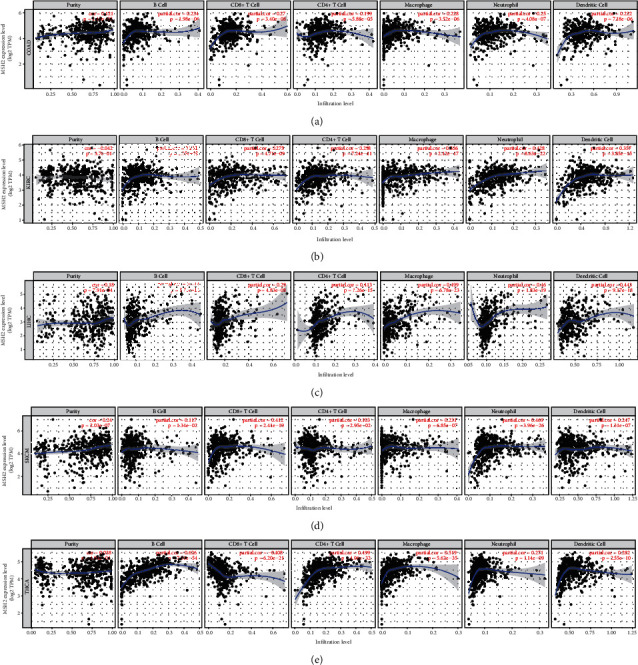
Correlation analysis between MSH2 expression and tumor immune infiltrating cells in pan-cancer. (a) Correlation analysis between expression levels of MSH2 and tumor immune infiltrating cells in COAD. (b) Correlation analysis between expression levels of MSH2 and tumor immune infiltrating cells in KIRC. (c) Correlation analysis between the expression level of MSH2 and tumor immune infiltrating cells in LIHC. (d) Correlation analysis between expression levels of MSH2 and tumor immune infiltrating cells in SKCM. (e) Correlation analysis between the expression level of MSH2 and tumor immune infiltrating cells in THCA.

We obtained the correlation coefficient between MSH2 expression level and immune cell infiltration level in 39 cancer types through TIMER database and selected MSH2 expression level and B cells, CD4+ T cells, CD8+ T cells, dendritic cells, macrophages, and neutrophils which related to cancers for analysis. The results showed that in COAD, the expression of MSH2 and B cells (*R* = 0.234, *P* < 0.001), CD4+ T cells (*R* = 0.199, *P* < 0.001), CD8+ T cells (*R* = 0.27, *P* < 0.001), macrophages (*R* = 0.228, *P* < 0.001), neutrophils (*R* = 0.25, *P* < 0.001), and dendritic cells (*R* = 0.222, *P* < 0.001) infiltration levels was significantly positively correlated; in KIRC, the expression of MSH2 and B cells (*R* = 0.261, *P* < 0.001), CD4+ T cells (*R* = 0.298, *P* < 0.001), CD8+ T cells (*R* = 0.275, *P* < 0.001), macrophages (*R* = 0.386, *P* < 0.001), neutrophils (*R* = 0.428, *P* < 0.001), and dendritic cells (*R* = 0.357, *P* < 0.001) infiltration levels was significantly positively correlated; in LIHC, the expression of MSH2 related to B cells (*R* = 0.388, *P* < 0.001), CD4+ T cells (*R* = 0.413, *P* < 0.001), CD8+ T cells (*R* = 0.29, *P* < 0.001), macrophages (*R* = 0.499, *P* < 0.001), neutrophils (*R* = 0.460, *P* < 0.001), and dendritic cells (*R* = 0.443, *P* < 0.001) infiltration levels was significantly positively correlated; in SKCM, the expression of MSH2 related to B cells (*R* = 0.117, *P* = 0.013), CD4+T cells (*R* = 0.103, *P* = 0.030), CD8+T cells (*R* = 0.412, *P* < 0.001), macrophages (*R* = 0.231, *P* < 0.001), neutrophils (*R* = 0.469, *P* < 0.001), and dendritic cells (*R* = 0.247, *P* < 0.001) infiltration levels was significantly positively correlated; in THCA, the expression of MSH2 related to B cells (*R* = 0.626, *P* < 0.001), CD4+T cells (*R* = 0.499, *P* < 0.001), macrophages (*R* = 0.519, *P* < 0.001), neutrophils (*R* = 0.271, *P* < 0.001), and dendritic cells (*R* = 0.282, *P* < 0.001) infiltration levels was significantly positively correlated, and CD8+ T cell (*R* = −0.408, *P* < 0.001) infiltration levels were significantly negatively correlated (Figure 4).

### 3.4. MSH2 Is Associated with Tumor Microenvironment in Pan-Cancer

**Figure 5:**
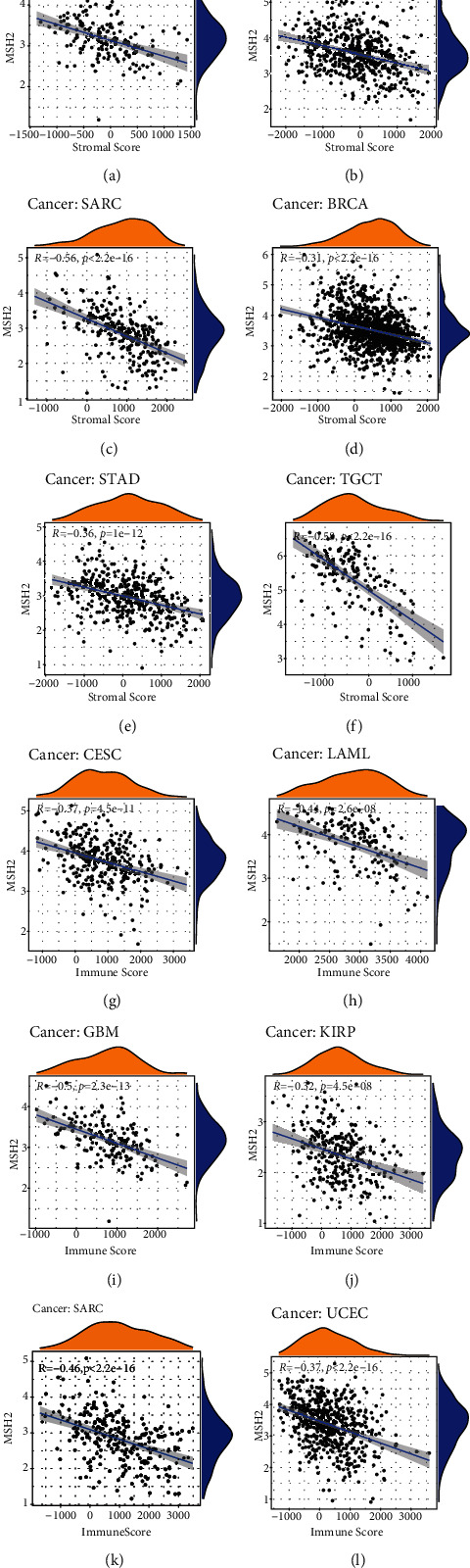
Correlation analysis of MSH2 expression and tumor microenvironment in pan-cancer. (a) Correlation between MSH2 expression and stromal score in GBM. (b) Correlation between MSH2 expression and stromal score in LUSC. (c) Correlation between MSH2 expression and stromal score in SARC. (d) Correlation between MSH2 expression and stromal score in BRCA. (e) Correlation between MSH2 expression and stromal score in STAD. (f) Correlation between MSH2 expression and stromal score in TGCT. (g) Correlation between MSH2 expression and immune score in CESC. (h) Correlation between MSH2 expression and immune score in LAML. (i) Correlation between MSH2 expression and immune score in GBM. (j) Correlation between MSH2 expression and immune score in KIRP. (k) Correlation between MSH2 expression and immune score in SARC. (l) Correlation between MSH2 expression and immune score in UCEC.

We use the R software package estimate to calculate the stromal score and immune score of 33 cancers and analyze the relationship between the expression level of MSH2 and these two scores. The results of the study showed that the top six tumors with the most significant correlation between MSH2 and stromal score were GBM (*R* = −0.45, *P* < 0.001), LUSC (*R* = −0.3, *P* < 0.001), SARC (*R* = −0.56, *P* < 0.001), BRCA (*R* = −0.31, *P* < 0.001), STAD (*R* = −0.36, *P* < 0.001), and TGCT (*R* = −0.58, *P* < 0.001) (Figures 5(a)–5(f)); the top six tumors with the most significant correlation between MSH2 and immune score are CESC (*R* = −0.37, *P* < 0.001), LAML (*R* = -0.44, *P* < 0.001), GBM (*R* = −0.50, *P* < 0.001), KIRP (*R* = −0.32, *P* < 0.001), SARC (*R* = −0.46, *P* < 0.001), and UCEC (*R* = −0.37, *P* < 0.001) (Figure 5(g)–5(l)).

### 3.5. The Expression of MSH2 in Pan-Cancer Is Related to Tumor Mutation Burden and Microsatellite Instability

**Figure 6:**
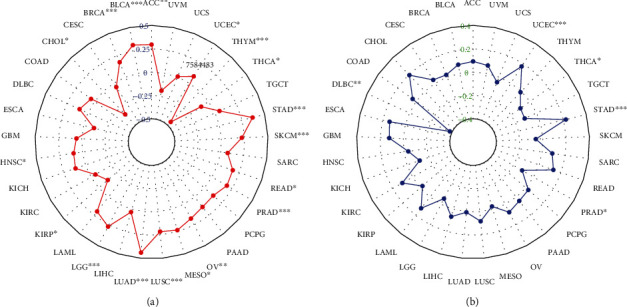
The correlation of MSH2 expression with TMB and MSI in pan-cancer. (a) Correlation between TMB and MSH2 expression. (b) Correlation between MSI and MSH2 expression.

TMB and MSI are considered as important factors inducing tumor occurrence and development. The analysis of correlation between MSH2 expression and TMB and MSI in 33 common cancers reflected that the expression of MSH2 in ACC, BLCA, BRCA, HNSC, LGG, LUAD, LUSC, MESO, OV, PRAD, READ, SKCM, STAD, and UCEC was significantly positively correlated with TMB. However, in CHOL, KIRP, THCA, and THYM, the expression of MSH2 was significantly negatively correlated with TMB (Figure 6(a)). The expression of MSH2 in STAD and UCEC was significantly positively correlated with MSI. On the contrary, the expression of MSH2 in THCA, PRAD, and DLBC showed significantly negative correlation with MSI (Figure 6(b)).

## 4. Discussion

Mismatch repair proteins are composed of multiple DNA base mismatch proteins that specifically repair DNA bases, which play an important role in maintaining the fidelity and stability of the genome and avoiding or reducing mutations in the process of gene coding [22]. MSH2 is an essential part of the DNA mismatch repair system for DNA damage repairment [23, 24]. According to the previous reports, the expression MSH2 was increased in oral squamous cell carcinoma and decreased in breast and gastric cancer [19, 25, 26]. MSH2 may have a functional consequence in different types of cancer, which is worthy of our further study.

The results of this study showed that compared with other tissues, MSH2 expression level is the highest in bone marrow tissue. Normally, bone marrow hyperplasia is active, which may lead to relatively high levels of MSH2 expression. Through analysis of the TCGA database, we found that MSH2 is highly expressed in a variety of cancer types when compared with the corresponding normal tissues. It has been reported that MSH2 expression is increased in low-grade and high-grade urothelial malignancy [27]. In addition, MSH2 was overexpressed in patients with colon cancer and oral squamous cell carcinoma [19, 28]. The research results showed above agreeing with our conclusions. Malik et al. proposed that in the Pakistani population, MSH2 deficiency may lead to the occurrence and development of breast cancer [25]. This result was inconsistent with our current results. The current study demonstrated that the expression of MSH2 in gastric cancer tissues was significantly reduced, especially in poorly differentiated gastric cancer when compared with normal gastric mucosal tissue [26]. The expression of MSH2 may indicate the advanced stages and negative prognosis of gastric cancer. This result may also be of relevance for different sample sources or the heterogeneity of the tumor. In this study, we demonstrated the prognostic value of MSH2 in pan-cancer. Kaplan-Meier analysis showed that high expression of MSH2 is associated with poorer OS in patients with ACC, LGG, LIHC, MESO, PAAD, SARC, and UCEC. In contrast, high expression of MSH2 was associated with a positive prognosis for patients with KIRC, STAD, and THYM. MLH1/MSH2 served as an independent prognostic and predictive factor for outcome of stage II/III sporadic colorectal cancer [29]. When receiving platinum-based chemotherapy, patients with low-expressing MSH2 bladder cancer had an inferior survival [30]. In short, these findings indicated that MSH2 can potentially work as a prognostic biomarker for pan-cancer.

Tumor cells, fibroblasts, immune cells, and the extracellular matrix are important components of the tumor microenvironment, which significantly affect the diagnosis and treatment of tumors. According to reports, tumor-infiltrating immune cells have an important impact on the occurrence and development of tumors which antagonizing or promoting the occurrence and development of tumors [31]. This study found that contrary to the negative correlation between the expression of MSH2 and CD8+T Cell in THCA, the expression of MSH2 was significantly positively correlated with the infiltration of six immune cells in COAD, KIRC, LIHC, and SKCM. MSH2 protein stimulated the proliferation of *γδ* T cells in peripheral blood mononuclear cells. *γδ* T cells were a small part of T lymphocytes and played an important role in tumor surveillance [32]. Existing evidence showed that MSH2 is overexpressed in pancreatic cancer cells and can be used as a CD4+ helper T cell antigens for the immunotherapy of patients with pancreatic cancer [33]. In LUAD, high expression of MSH2 was significantly correlated with CD8+ T cell infiltration [34]. In addition, the loss of MMR protein expression was related to the selective downregulation of human leukocyte antigen class I antigens, which contributes to the immune escape of endometrial carcinomas [35]. We also found that in GBM, LUSC, SARC, BRCA, STAD, and TGCT, MSH2 expression was significantly negatively correlated with stromal score; in CESC, LAML, GBM, KIRP, SARC, and UCEC, MSH2 expression and immune score were significantly negatively related. In short, these results indicate that the abnormal expression of MSH2 is closely related to the immune infiltration of tumor cells, which may change the immune microenvironment of tumor and patients outcome.

TMB is a promising biomarker for pan-cancer prediction, leading immunotherapy into the era of precision medicine [36]. In LUAD, the increase in MSH2 expression was significantly positively correlated with TMB [34]. Studies have shown that high nonsynonymous TMB was a good prognostic factor for patients with non-small-cell lung cancer [37]. TMB was associated with the survival rate of patients with various cancer types treated with immune checkpoint inhibitor (ICI) [38]. TMB and MSI are important biomarkers of ICI, and there is a certain correlation between the two. Studies have shown that high MSI and high TMB occur simultaneously in gastrointestinal cancers such as stomach adenocarcinoma, duodenum adenocarcinoma, and small intestine adenocarcinoma [39]. Our research showed that the expression level of MSH2 was significantly correlated with TMB in ACC, BLCA, BRCA, HNSC, LGG, LUAD, LUSC, MESO, OV, PRAD, READ, SKCM, STAD, UCEC, CHOL, KIRP, THCA, and THYM; the expression level of MSH2 was significantly correlated with STAD, UCEC, THCA, PRAD, and DLBC. This indicated that the expression level of MSH2 may affect the response of patients with immune checkpoint suppression therapy by affecting the TMB and MSI of cancer. However, further research need to determine whether MSH2 can be used as a predictor of the efficacy of immunotherapy in related cancer types. In conclusion, the results of this study provide clues to the link between MSH2 and cancer immunity.

## 5. Conclusion

This study revealed the role of abnormal expression of MSH2 in the occurrence and development of pan-cancer through comprehensive by bioinformatics methods and indicated that MSH2 expression may mediate immune infiltration and affect the prognosis of pan-cancer patients. MSH2 can emerge as a potential biomarker for cancer diagnosis and prognosis, providing a new direction for exploring the pathogenesis of pan-cancer. However, this study still has certain limitations. First of all, this research is relying on public databases and short of verification by experiments. Secondly, MSH2 is highly expressed in a variety of cancers and related to poor prognosis, but the specific mechanism of this effect still needs further investigation. The expression of MSH2 also has a certain correlation with tumor microenvironment, TMB, and MSI, however lack of data to verify its correlation. In the future, we will continue to explore the mechanism of action of MSH2 in different cancer types at the cellular or molecular level based on the results of this study.

## Data Availability

The data used to support the findings of this study are available from the corresponding author upon request.
